# Teleultrasound: Historical Perspective and Clinical Application

**DOI:** 10.1155/2015/306259

**Published:** 2015-02-24

**Authors:** Adilson Cunha Ferreira, Edward O'Mahony, Antonio Hélio Oliani, Edward Araujo Júnior, Fabricio da Silva Costa

**Affiliations:** ^1^Department of Obstetrics and Gynaecology and Department of Perinatal Medicine, Pregnancy Research Centre, Royal Women's Hospital, University of Melbourne, Melbourne, VIC 3207, Australia; ^2^School of Medicine, University of São José do Rio Preto, São José do Rio Preto, SP, Brazil; ^3^Monash Ultrasound for Women, Melbourne, VIC, Australia; ^4^Department of Obstetrics, Paulista School of Medicine-Federal University of São Paulo (EPM-UNIFESP), 05303-000 São Paulo, SP, Brazil

## Abstract

The health care of patients in rural or isolated areas is challenged by the scarcity of local resources, limited patient access to doctors and hospitals, and the lack of specialized professionals. This has led to a new concept in telemedicine: teleultrasonography (or teleultrasound), which permits ultrasonographic diagnoses to be performed remotely. Telemedicine and teleultrasonography are effective in providing diagnostic imaging services to these populations and reduce health care costs by decreasing the number and duration of hospitalizations and reducing unnecessary surgical procedures. This is a narrative review to present the potential clinical applications of teleultrasonography in clinical practice. The results indicate that although barriers persist for implementing teleultrasonography in a more universal and routine way, advances in telecommunications, Internet bandwidth, and the high resolution currently available for portable ultrasonography suggest teleultrasonography applications will continue to expand. Teleultrasound appears to be a valuable addition to remote medical care for isolated populations with limited access to tertiary healthcare facilities and also a useful tool for education and training.

## 1. Introduction

Under the WHO definition of telemedicine, “the delivery of health care services, where distance is a critical factor,” it is intended for the exchange of valid information for the diagnosis, prevention, and treatment of disease and for the continuing education of health service providers, as well as for research and evaluation purposes [1]. Ultrasonography is very useful diagnostic tool because it is a noninvasive, generally nonexpensive, and highly portable method that does not use ionizing radiation [2]. However, generating and interpreting ultrasound images are highly operator-dependent. As a result, performance and interpretation of these examinations have traditionally been limited to medical specialists [3, 4]. Although some remote areas have access to basic primary care services, including X-rays and ultrasound, they frequently lack specialized radiologists and ultrasonographers [5]. This lack of experienced doctors or qualified technicians has led to a new concept in telemedicine: teleultrasonography (or teleultrasound), which permits ultrasonographic examinations to be performed remotely in a synchronous (real-time) or asynchronous fashion. Over the past decade, research on teleultrasonography has evolved incrementally, especially with regard to technology, which is why most of the work has been published in technical engineering and medical informatics journals. However, few studies have been published on training doctors and technicians from remote or isolated areas.

This paper presents a narrative review of the potential clinical applications of teleultrasound in clinical practice.

## 2. Historical Perspectives

The committee formed by the collaboration between ACR, American College of Radiology, and NEMA, National Electrical Manufacturers Association in 1983, aimed to solve the problem of the Babel of files formats, images, and information generated by different manufacturers and equipment and to define the parameters for the transmission of distance medical images in an asynchronous way. From this point onwards, several studies were carried out in order to prove the diagnostic accuracy of this type of transmission, which is well established today [6–8].

Among the different areas of teleradiology, teleultrasonography is potentially the safest and least expensive area because it does not utilize ionizing radiation and is affordable [3, 4]. Investigators in the United States, the United Kingdom, Canada, and Australia were the pioneers in this research and in carrying out pilot studies [5–9]. The majority of these projects were based on the use of hardware and software packages which required high speed broadband connections and were associated with a high cost of implementation [5].

## 3. Transmission Modes

The asynchronous method is noninteractive. In this mode, data are collected, stored, and then forwarded to a sonologist for interpretation. This system can capture and store images as well as audio and text. This mode eliminates the need for the doctor and patient to be present at the same time in the same place and is widely used in countries where technicians instead of doctors perform ultrasound examinations [7, 10]. It is also widely used in university hospitals where trainees perform the examination and save the images, which are then evaluated together with the supervising physician [7, 11]. This type of service increases population access to services that are not locally available and is currently the most widespread mode of using teleultrasonography [11, 12]. In the synchronous mode, data transmission takes place in real time, that is, while the examination is being performed. This mode enables more experienced doctors to remotely supervise any person who is operating the ultrasound equipment, including technicians, medical residents, and personnel in training [12]. This system reduces the need for face-to-face consultations and allows for earlier detection of problems, which may further reduce the costs associated with the delocalization of specialized personnel. In the medical field, this modality involves a real-time interaction between the patient, the person operating the ultrasound equipment, and the interpreting specialist who will report the final diagnosis. This mode is not only possible but is well accepted by the physicians and patients involved. With the widespread use of the Internet across the world and increased communication via fixed or wireless broadband Internet access, the synchronous mode should become an important tool for clinical practices and educational purposes [13–15].

## 4. Research Categories and Themes

**Figure 1:**
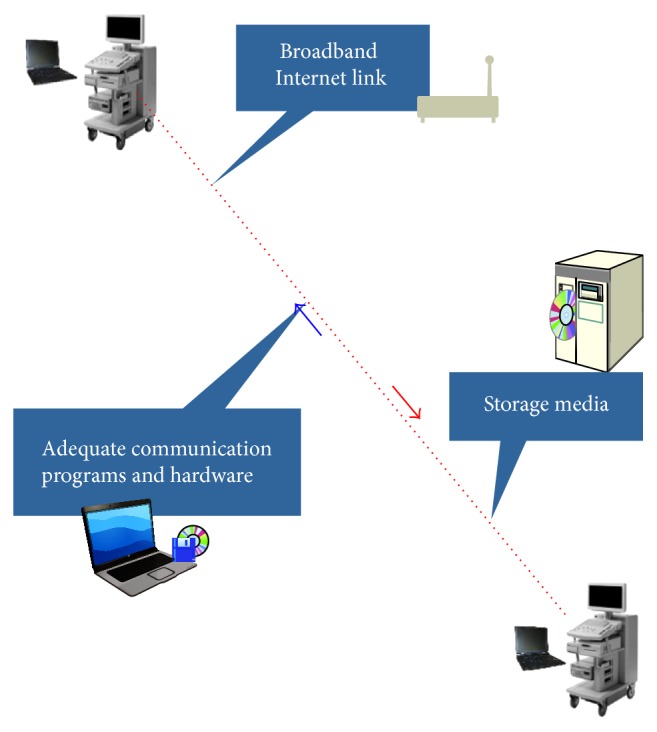
Illustrative description of the system utilized to real-time transmission of video clips and images between different centers.

Research on teleultrasonography can be divided into studies that use synchronous (real-time) (Figure 1) and asynchronous (store-and-forward) transmission. Within these two categories of research, there are three recurring themes that are relevant to the successful implementation of a teleultrasonography system: (a) the quality of the transmitted images; (b) clinical applications; (c) the nontechnical and technical barriers to implementation [4].

Opinions in the literature are divided with regard to the transmission system involved in teleultrasonography. Some authors consider that good image quality can only be obtained with the use of asynchronous transmission, which, in addition to good diagnostic accuracy, allows for the training and professional supervision to produce a satisfactory level of clinical competence [6–8, 10, 12]. Other studies have sought to demonstrate the accuracy of the teleultrasonography performed in real time between a tertiary center and a remote area. The authors argue that the image quality was not very clear when teleultrasonography first started but that current telecommunication and image compression technologies have made high-quality synchronous and asynchronous transmissions feasible [5, 16]. Other authors argue in favour of real-time transmissions because the asynchronous mode only allows images and videos to be stored for future analysis and their interpretation may be incomplete or diagnostically inaccurate if some important information is missing and cannot be recovered [17]. However, the increasingly routine use of real-time transmission is still associated with high costs, and various studies have proposed alternatives to reduce these costs [5, 18–20].

## 5. Image Quality

Studies focused on the quality of transmitted images have attempted to determine the minimum bandwidth required, the most efficient processing signal, and the optimal compression system to generate an image with high diagnostic value [19]. The first studies on the quality of image in teleultrasound examined the transmission with the use of bandwidths such as 128 kbit/s, 256 kbit/s, and 384 kbit/s and suggested that the larger the bandwidth, the greater the amount of information received by the observer, which resulted in a more accurate diagnosis [21–25].

**Figure 2:**
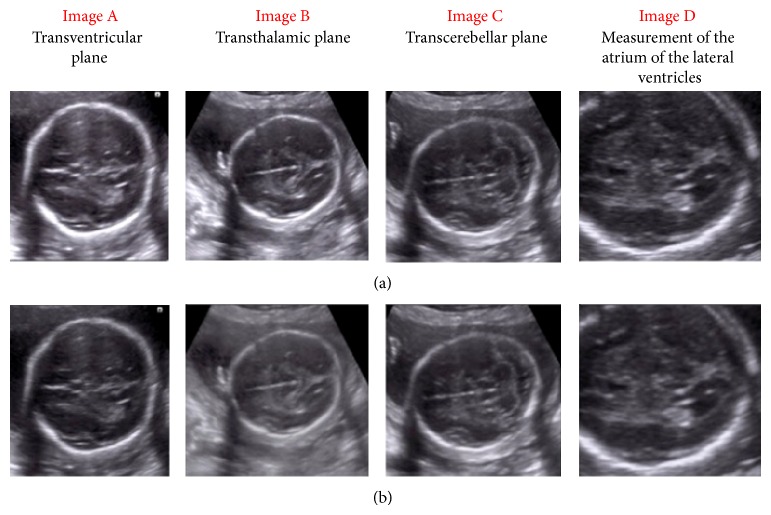
Transventricular, transthalamic, transcerebellar, and measurement of the atrium of the lateral ventricles; (a) original and (b) transmitted image planes of fetal central nervous system.

Whereas some researchers have evaluated the quality of transmitted images using analyses made by other radiologists, other researchers have measured image degradation using a careful analysis of the Fourier spectrum and contrast measurements [20]. Some studies have used a robotic arm for real-time transmissions. The results indicate that, although the duration of such tests is longer than that required for an in-person examination, the method could provide diagnostic information that is not available in remote or inaccessible areas [26, 27] (Figure 2).

## 6. Advances in Real-Time Transmission

More recently, a new type of image transmission has emerged under the name of Remote Task Scale, where the expert sees the images transmitted in real time with loss of quality but remotely receives the tests' recorded sequence of images with total quality. This modality can be implemented with the use of broadband technology of medium and high speed and with the use of streaming video [19, 20, 28].

With the advancement of open networks, image compression technology, and bandwidth levels in many countries, including developing countries, many locales have bandwidth that exceeds the minimum bandwidth recommended for transmission in the initial studies published a decade ago on high-quality synchronous and asynchronous transmissions. Advances in telecommunications and signal processing and the relatively high resolution currently available for portable ultrasound equipment greatly increase the potential clinical applications of teleultrasonography. The remaining technical and logistical barriers include the availability of telecommunications in the desired areas and the training of medical professionals involved in the transmission, reception, and analysis of the images [4, 29].

## 7. Training and Education

According to the World Health Organization (WHO), diagnostic imaging is a necessary procedure for accurately treating at least 25% of patients worldwide. However, there is currently a lack of imaging services in vast areas of the world, especially in developing countries [1]. Where they do exist, these services are often of poor quality, resulting in misdiagnoses or nondiagnoses [20]. This is because the diagnostic quality of ultrasound images is extremely dependent on the physician and requires a certain level of expertise to obtain all the image planes necessary and interpret their meaning [11, 17]. Thus, even having the appropriate technology at their disposal, many patients in geographically or socially isolated areas may remain disadvantaged by the lack of a trained professional to operate them [17]. In reality, many small medical centers and isolated locations do not have well-trained sonographers to perform the initial assessments that provide an accurate diagnosis. However, although there have been advances in research in the field of teleultrasonography, studies addressing the training of medical or technical personnel are scarce. In our review, only 5% of the literature on teleultrasonography evaluated education and training of operators. Yet these studies did not systematically quantify the degree to which the professionals involved had improved.

A study in Italy reported that, in relation to the use of teleultrasonography, at least one month of didactic and hands-on training should be performed by operators to ensure that they have acquired sufficient technical competence [30]. Other authors recommended that training should be performed in the workplace of the local doctors by radiologists or sonologists who will evaluate and interpret the images [10, 13, 24]. A more recent study proposed that remote guidance by a specialist be given in real time to instruct inexperienced doctors in acquiring and visualizing the appropriate planes during examinations. The authors considered this educational tool superior to verbal instruction while training doctors at a distance because it enables new skills to be acquired in half the time required using traditional educational practices [15].

Currently, there are no standard training protocols for sonographers in remote locations. To ensure a greater degree of quality control, the images produced locally should be continuously examined via the Internet by competent radiologists and sonologists to ensure that they are following the proper scanning protocols and that the images contain sufficient diagnostic value [3, 4]. The teleultrasonography and other forms of telemedicine continue to grow; it is important that high quality standards be maintained, or its use may be detrimental to the population. The establishment of a framework to address and examine telemedical errors has been proposed to ensure high levels of quality and safety [31]. Regarding teleultrasonography, gaps in quality include inappropriate scanning protocols, improper scanning technique, inadequate image collection, incomplete examinations, false diagnoses, and nondiagnoses [3, 4, 15].

## 8. Clinical Utility

A significant portion of teleultrasonography research has focused on its use on an outpatient basis with emphasis on the areas of obstetrics and fetal medicine, especially to confirm pregnancy, monitor fetal growth, and evaluate pregnancy related complications such as placenta previa or placenta accreta.

An increasing number of new applications for teleultrasonography have included investigations of almost every organ and system in various medical fields, including the diagnosis of clinical or surgical conditions, examining severely ill patients and guided procedures [2, 15]. In recent years, many systems have been developed for the paramedic care of trauma patients, both at the scene of an accident and during transport to the hospital, allowing medical teams to adequately prepare to perform required emergency procedures before the patient's arrival [17]. Despite the persistence of barriers in implementing teleultrasonography more broadly and routinely in the initial care administered at the site of trauma, the advances in telecommunications, Internet signal processing, and the high resolution currently available for portable ultrasound equipment increase the scope of applications for teleultrasonography [29]. The remaining technical and logistical barriers are being solved on a daily basis and include improving the availability of telecommunications in desired areas, the reception and analysis of the images, and the training of medical professionals involved in transmission [4].

Several studies on teleultrasonography in remote areas of Australia and Canada have demonstrated its usefulness in gestational and fetal heart examinations [2]. However, most of these studies demonstrated a good correlation between diagnoses made locally and those made remotely [7, 21, 22, 24, 32–34]. One example of this correlation was reported in studies conducted in Queensland, Australia. This state, which had a population of 3.3 million, 55% of whom lived in rural areas, only had two specialized fetal-maternal echography centers, which were located at universities in the capital, Brisbane. The implementation of a teleultrasonography service showed good diagnostic accuracy and identified all of the existing fetal abnormalities in the cases examined. Clinicians for these patients stated that, in the absence of teleultrasonography, they would have sent 1/3 of these patients to the closest specialized ultrasonography center, which was located 1500 km away [13, 14, 35].

A similar study was conducted to review the usefulness, accuracy, and benefits of teleultrasonography in primary care hospitals in South Dakota (USA) that care for neonates with suspected congenital heart disease. Two primary hospitals established a telemedicine link with a tertiary center. A pediatric cardiologist interpreted the neonatal teleultrasound images, and the results and recommendations of the specialist were immediately reported to the attending physicians. The study concluded that teleultrasonography could accurately distinguish between neonates who needed immediate cardiac care and those with less critical heart disease, thereby providing immediate diagnoses and appropriate care [36].

## 9. Future Directions

Although it is clear that ultrasonography is an easily adaptable imaging technology, especially for use in developing countries, the mere presence of the equipment does not indicate improvements in patient care [17]. With the continuous development and democratization of the Internet and other information technologies, teleultrasonography has the potential to assist in the provision of specialist services to remote locations. In addition to typical teleultrasonography applications in routine clinical practice, emergency interventional procedures guided by teleultrasonography, such as thoracocentesis, pericardiocentesis, and paracentesis, are already being used experimentally in intensive care units. The routine use of these and other innovations should result in overall improvements in patient care in remote and isolated regions [17].

The future for the application of teleultrasound is potentially enormous, both for the developed countries and for the developing ones. Several efforts have been made in order to increase the simplification of the ultrasound equipment, increasing their portability. These advances open new areas of potential applications [37]. In the future, these technologies will be common place and will be first-use technologies, providing rapid and accurate diagnoses and improving the quality of patient care, especially in remote communities [11]. These technologies have led some scholars to consider the tool of teleultrasonography as the “stethoscope of the future” [38].

The routine use of portable ultrasound equipment allied to technological advances may be used for educational purposes in practically any place in the world, providing training of quality and permanent updating for ultrasonography professionals living in remote or needy regions. These innovations combined with educational strategies and the introduction of strict training protocols for health care workers, primarily but not exclusively in remote communities, will allow teleultrasonography to be definitively established as an effective tool in health care and an important tool in educating and training individuals working with ultrasonography. With increased use of ultrasonography, the need for education and training of users becomes clear.

Notably, teleultrasonography has been studied very little for its use in the continuing education of health care providers. Future studies should be conducted not only to quantify the results of patient care but also to measure the degree of training of the professionals involved because performing supervised ultrasound examinations can be used as a tool for a second opinion, encouraging continuous and gradual improvement.

## 10. Barriers to the Implementation of Clinical Teleultrasound

In the 90s, Wooton summarized the critical questions that needed to be approached in order to develop a plan for the implementation of telemedicine: to evaluate the necessary structural changes to incorporate technology, develop an education and training process and formulate guidelines, quality control, and continuous audit [39]. These questions also apply to teleultrasound and are up-to-date even nowadays.

Teleultrasound is a specific type of telemedicine that uses the technological advances for the remote interpretation of ultrasound images. Nevertheless, software, hardware, and/or video-conferences platforms that are necessary for the transmission of images are still highly costly which is one of the main barriers for its implementation in the developing countries [37, 40].

Secondly, although the technological barriers for the development of teleultrasound are continuously disappearing, the nontechnological ones still persist. These barriers include deficits in training and operational protocols, complexity in the use of equipment, and concerns regarding the security and, by extension, the confidentiality of electronically transmitted information [3, 4, 41, 42].

Table 1 summarizes the main findings of published articles about teleultrasound.

## 11. Conclusions

Teleultrasound is a valuable addition to remote medical care for isolated populations with limited access to tertiary healthcare facilities. As some studies point out, the portability and the low cost of the equipment frequently make ultrasonography the only modality of image test available in remote places. The implementation of teleechography in remote or needy places allows for more timely detection of problems, facilitates the obtaining of a second opinion, and reduces the costs of moving. Educational strategies for health agents in remote or needy locations must be continuously researched and developed with the purpose of standardizing training protocols and providing quality assistance to these populations.

## Figures and Tables

**Table 1:** Main findings about the published articles about teleultrasound.

Reference number	Author	Title and journal	Year	Main findings
[1]	World Health Organization–WHO	Telemedicine: opportunities and developments in Member States. Reports on the Second Global Survey on eHealth 2009. HYPERLINK “http://www.who.int/goe/publications/goe_telemedicine_2010.pdf”	2009	(1) Teleradiology currently has a 33% rate of established service provision globally. (2) While developing countries are more likely to consider resource issues such as high costs, underdeveloped infrastructure, and lack of technical expertise to be barriers to telemedicine, developed countries are more likely to consider legal issues surrounding patient privacy and confidentiality, competing health system priorities, and a perceived lack of demand to be barriers to telemedicine implementation. (3) Following the analysis of the survey results, WHO recommends steps member states can take to capitalize on the potential of ICTs. One such step is creation of national agencies to coordinate telemedicine and eHealth initiatives, ensuring they are appropriate to local contexts, cost-effective, consistently evaluated, and adequately funded as part of integrated health service delivery. Ultimately telemedicine initiatives should strengthen rather than compete with other health services.

[2]	Law and Macbeth	Ultrasound: From Earth to Space. MJM 2011; 13(2): 59–65.	2011	Ultrasound is a well-proven diagnostic modality on Earth and is becoming increasingly useful in space. Ultrasound shows much promise in benefitting both astronauts and patients on Earth.

[3]	Sutherland et al.	A comparison of telesonography with standard ultrasound care in a rural Dominican clinic. J Telemed Telecare. 2009; 15(4); 191–5.	2009	The pilot study demonstrated that store-and-forward telesonography reduced time of diagnosis and increased the continuity of care compared to the usual ultrasound referral system in the region of the Dominican Republic which was studied.

[4]	Sutherland et al.	Telesonography: foundations and future directions. J Ultrasound Med. 2011; 30(4): 517–22.	2011	Future projects may use telesonography to supplement the training of health care providers in remote locations in an effort to establish permanent sonography services for their respective communities.

[5]	Popov et al.	The feasibility of realtime transmission of sonographic images from a remote location over lowbandwidth Internet links: a pilot study. AJR Am J Roentgenol. 2007; 188(3): 219–22.	2007	Real-time transmission of sonographic images over low bandwidth Internet links offers the potential for sonography to be performed at a remote underdeveloped region and interpreted in real time at a distant site by trained radiologists, thereby extending the presence of physicians in virtual space.

[6]	Hersh et al.	The evidence base of telemedicine. J Telemed Telecare. 2006; 12(Suppl 2): S1-2.	2006	

[7]	Ferrer-Roca et al.	Tele-virtual sonography. J Perinat Med. 2006; 34(2): 123–9.	2006	(1) 3D reconstruction could reduce multiple explorations due to image constrains such as suboptimal fetal positioning among others. (2) Virtual sonography was important to reach confidence on distant diagnosis. (3) It was also considered a tool for offline local review of nontrained sonographer acquisitions.

[8]	Kasmai	Realtime Telesonography: Vision or Reality? Ultrasound. 2006; 14(3): 152–4.	2006	It is now possible to configure and set up a streaming telesonography service with minimum cost and effort.

[9]	Afset and Lunde	Tele-echocardiography. Education in echocardiography via video conferences. Tidsskr Nor Laegeforen. 1994; 114(10): 1175–8.	1994	Teleechocardiography is a method that is suitable for basic training in echocardiography and the diagnostic precision is sufficient for the method to be applied clinically.

[43]	Afset et al.	Accuracy of routine echocardiographic measurements made by an inexperienced examiner through tele-instruction. J Telemed Telecare. 1996; 2(3): 148–54.	1996	The reproducibility and accuracy of routine echocardiographic measurements made by an inexperienced doctor using teleinstruction were comparable to those observed in reproducibility studies made under normal examination conditions. There were no systematic measurement errors. Teleinstructed echocardiography is also an excellent educational tool, allowing an inexperienced examiner gradually to take responsibility for the local echocardiographic service.

[44]	Trippi et al.	Emergency echocardiography telemedicine: an efficient method to provide 24-hour consultative echocardiography. J Am Coll Cardiol. 1996; 27(7): 1748–52.	1996	

[45]	Mulholland et al.	Application of a low cost telemedicine link to the diagnosis of neonatal congenital heart defects by remote consultation. Heart. 1999; 82(2): 217–21.	1999	Transmitted images were of sufficient quality to allow confirmation or exclusion of major congenital heart disease. The telemedicine link facilitated early diagnosis and initiation of appropriate management in patients with complex congenital heart disease and avoided the need for transfer in those where significant congenital heart disease was excluded.

[21]	Wootton et al.	The effect of transmission bandwidth on diagnostic accuracy in remote fetal ultrasound scanning. J Telemed Telecare. 1997; 3(4): 209–14.	1997	A study which found that, although there were no perceived differences in technical quality between recordings transmitted at 384 or 1920 kbit/s, diagnostic accuracy was marginally worse at the lower bandwidth. This suggests that the higher bandwidth conveys more detail and information to the observer, which in turn enables more accurate diagnosis. However, further work is required before a definitive choice can be made about the optimum transmission bandwidth for remote fetal ultrasound studies.

[22]	Hussain et al.	Evaluation of a training and diagnostic ultrasound service for general practitioners using narrowband ISDN. J Telemed Telecare. 1999; 5(Suppl1): S95–9.	1999	A pilot study which demonstrated that store-and-forward images are far superior to hard-copy images for technical quality.

[23]	Brebner et al.	The diagnostic acceptability of low-bandwidth transmission for tele-ultrasound. J Telemed Telecare. 2000; 6(6): 335–8.	2000	The quality of dynamic ultrasound images transmitted at 384 kbit/s was diagnostically acceptable but was unsatisfactory at 128 kbit/s.

[10]	Hussain et al.	The feasibility of telemedicine for the training and supervision of general practitioners performing ultrasound examinations of patients with urinary tract symptoms. J Telemed Telecare. 2004; 10(3): 180–2.	2004	Forwarded images were superior to hard-copy images.

[11]	Adambounou et al.	System of telesonography with synchronous teleconsultations and nchronous telediagnoses. Med Sante Trop. 2012; 22(1): 54–60.	2012	A successful pilot trial of a low cost synchronous teleultrasound system in a developing country.

[12]	Meuwly	Telesonography-Modern Solutions for an old Question. Ultraschall in Med 2010; 31(4): 421–3.	2010	As volume US acquisition seems to be rather independent of the skill of the local operator, asynchronous remote interpretation of reconstructed images from volume data sets appears to be a well-appropriate technical solution for telesonography, provided that suitable workstations for postprocessing will be available.

[13]	Chan et al.	Randomized comparison of the quality of realtime fetal ultrasound images transmitted by ISDN and by IP video conference. J Telemed Telecare. 2002; 8(2): 91–6.	2002	We compared the quality of real-time fetal ultrasound images transmitted using ISDN and IP networks. There were no significant interobserver variations. The most significant variable affecting the mean score was the bandwidth used. IP transmission in a private (nonshared) network is an acceptable alternative to ISDN for fetal teleultrasound and one deserving further study.

[14]	Lewis	A tele-ultrasound needs analysis in Queensland. J Telemed Telecare. 2006; 11(Suppl 2): S61–4.	2006	A teleultrasound needs analysis in Queensland which found that approximately 10% of cases would have benefited from telesonography and that there was a strong preference for store and forward transmission.

[15]	Sheehan et al.	Expert visual guidance of ultrasound for Telemedicine. J Telemed Telecare. 2010; 16(2): 77–82.	2010	An inexperienced ultrasonographer can be significantly assisted by EVG compared to verbal instruction alone. This could be useful for telementoring in rural hospitals as well as for teaching, both in person and at a remote site.

[16]	Yoo et al.	Performance of a web-based, realtime, tele-ultrasound consultations system over highspeed commercial telecommunications lines. J Telemed Telecare.2004; 10: 175–9.	2004	A Web-based, real-time teleultrasound consultation system was designed and tested by radiologists. A bit rate of more than 0.6 Mbit/s, at 30 frames/s, is suggested as the threshold for the maintenance of diagnostic image quality.

[17]	Pian et al.	Potential Use of Remote Telesonography as a Transformational Technology in Underresourced and/or Remote Settings. Emergency Medicine International. 2013. Article ID 986160, 9 pages. HYPERLINK “http://dx.doi.org/10.1155/2013/986160”	2013	This paper summarizes the current literature surrounding the development of teleultrasound as a transformational technology and its application to underresourced settings.

[18]	Ferlin et al.	Tele-obstetric ultrasound: analysis of first-trimester ultrasound images transmitted in realtime. J Telemed Telecare. 2012; 18(1): 54–8.	2012	Teleobstetric ultrasound: analysis of first-trimester ultrasound images transmitted in real-time; the quality of images transmitted via the Internet through the use of low-cost software appeared suitable for screening for chromosomal abnormalities in the first trimester of pregnancy.

[19]	Paulus and Thompson	Inexpensive, realtime teleultrasound using a commercial, web-based video streaming device. J Telemed Telecare. 2012; 18: 185–8.	2012	System was feasible; response times increased with increasing distance.

[20]	Bassignani et al.	Review of technology: planning for the development of telesonography. J Digital Imaging. 2004; 17(1); 18–27.	2004	With less compression, the bit rate rises, and the only way the encoder can contain bit rate within the set bandwidth is by lowering frame rate or reducing image quality. Review the relevant technologies and industry standard components that will enable low-cost telesonography.

[24]	Chan et al.	Realtime fetal ultrasound by telemedicine in Queensland. A successful venture? J Telemed Telecare. 2001; 7(Suppl 2): 7–11.	2001	A real-time fetal teleultrasound consultation service in Queensland, which uses ISDN transmission at 384 kbit/s, 1500 km away. All significant anomalies and diagnoses have been confirmed. A crude cost-benefit calculation suggests that the teleultrasound service resulted in a net saving which enabled almost four times the number of consultations to be carried out.

[25]	O'Neill et al.	The design and implementation of an off-the-shelf, standards-based tele-ultrasound system. J Telemed Telecare. 2000; 6(Suppl 2): S52-3.	2000	A feasible DICOM system, synchronous.

[26]	Arbeille et al.	Use of a robotic arm to perform remote abdominal telesonography. AJR Am J Roentgenol. 2007; 188(4): 317–22.	2007	Robotic telesonography can be used for reliable diagnosis without moving the patient. No false diagnoses were made in this study. A bandwidth of 250 Kbps via integrated services digital network or satellite is required for reliable diagnosis.

[27]	Courreges et al.	Clinical trials and evaluation of a mobile, robotic tele-ultrasound system. J Telemed Telecare. 2005; 11 Suppl 1: 46–9.	2005	Feasibility of a robotic teleultrasound system.

[28]	Martini et al.	A Cross-Layer Approach for Wireless Medical Video Streaming in Robotic Teleultrasonography. IEEE Eng Med Bio Conference.2007 (EMBC 2007), Lyon, France, August 2007.	2007	Successful performance of video streaming in a robotic teleultrasonography system through a cross-layer approach based on tailor made controller structures is presented.

[29]	Fuentes	Remote interpretation of ultrasound images. Clin Obstet Gynecol. 2003; 46(4): 878–81.	2003	In the near future, the ability to transmit volume rendered images over standard phone lines will enhance the application of telesonography.

[30]	Cavina et al.	Telesonography: technical problems, solutions and results in the routine utilization from remote areas. Studies Health Technol Informatics. 2001; 81: 81–9.	2001	

[31]	Demiris et al.	To telemedically err is human. Joint Commission. J Quality Safe. 2004; 30(9): 521–7.	2004	To address patient safety and provide high-quality care, a framework for addressing and examining telemedical errors needs to be established.

[32]	Lagalla	Telecommunications, health and radiology: potential synergies for the new millennium. Radiol Med. 2001; 102(1-2): 14–9.	2001	Italian paper to highlight the potentialities and limitations in the use of teleradiology and to provide a set of recommendations/guidelines.

[33]	Soong et al.	The fetal tele-ultrasound project in Queensland. Aust Health Rev. 2002; 25(3): 57–73.	2002	We report on some of our practical experiences and difficulties in establishing such a service.

[34]	Arbeille et al.	Fetal-tele-ecography using a robotic arm and a satellite link. Ultrasound Obstet Gynecol. 2005; 26(3): 221–6.	2005	Teleechography using a robotic arm provides the main information needed to assess fetal growth and the intrauterine environment within a limited period of time.

[36]	Awadallah et al.	Tele-echocardiography in neonates: utility and benefits in South Dakota primary care hospitals. S D Med. 2006; 59(3): 97–100.	2006	A study of teleechocardiography to assess neonates with suspected congenital heart disease. Teleechocardiography accurately distinguished neonates who required tertiary cardiac care from those with less critical cardiac disease fostering prompt diagnosis and appropriate care while subjecting a minimal number of patients to costly emergency transport.

[37]	Crawford et al.	How to set up a low-cost teleultrasound capable videoconference system with wide applicability. Critical Ultrasound J. 2012; 4: 13.	2012	A functional remote telementored ultrasound (RTUS) system was constructed with a laptop computer wireless Internet and/or was tethered through a smartphone. The RTUS system allowed real-time mentored teleultrasound to be conducted from a variety of settings via VOIP transmissions. Numerous types of ultrasound examinations were conducted such as abdominal and thoracic examinations with a variety of users mentored who had previous skills ranging from none to expert. Internet connectivity was rarely a limiting factor, with competing logistical and scheduling demands of the participants predominating.

[38]	Su et al.	Application of Tele-Ultrasound in Emergency Medical Services. Telemed e-Health. 2008; 14(8): 816–24.	2008	This study describes the development of teleultrasound for prediagnosis in a medical emergency setting which will enhance prediagnosis options for on-duty emergency physicians; emergency medical technicians can also obtain instructions from on-duty physicians to enhance damage and disaster control ability in critical moments.

[39]	Wootton	Telemedicine in the National Health Service. J Roy Soc Med 1998; 91: 614–21.	1998	Having become technically and economically feasible, telemedicine deserves investigation by well-conducted research, which is adequately funded.

[40]	World Health Organization	Telemedicine: opportunities and developments in member states report. In Second Global Survey on eHealth Global Observatory for eHealth Series. 2011, vol 2. WHO Press, Switzerland.	2011	
